# Tyrosine decarboxylase activity of *Enterococcus mundtii*: new insights into phenotypic and genetic aspects

**DOI:** 10.1111/1751-7915.12402

**Published:** 2016-09-14

**Authors:** Veronica Gatto, Giulia Tabanelli, Chiara Montanari, Valentina Prodomi, Eleonora Bargossi, Sandra Torriani, Fausto Gardini

**Affiliations:** ^1^ Department of BiotechnologyUniversity of VeronaVeronaItaly; ^2^ Department of Agricultural and Food SciencesUniversity of BolognaCesenaItaly; ^3^ Interdepartmental Center for Industrial Agri‐food ResearchUniversity of BolognaCesenaItaly

## Abstract

Few information is available about the tyraminogenic potential of the species *Enterococcus mundtii*. In this study, two plant‐derived strains of *E. mundtii* were selected and investigated to better understand the phenotypic behaviour and the genetic mechanisms involved in tyramine accumulation. Both the strains accumulated tyramine from the beginning of exponential phase of growth, independently on the addition of tyrosine to the medium. The strains accumulated also 2‐phenylethylamine, although with lower efficiency and in greater extent when tyrosine was not added. Accordingly, the tyrosine decarboxylase (*tyrDC*) gene expression level increased during the exponential phase with tyrosine added, while it remained constant and high without precursor. The genetic organization as well as sequence identity levels of *tyrDC* and tyrosine permease (*tyrP*) genes indicated a correlation with those of phylogenetically closer enterococcal species, such as *E. faecium*,* E. hirae* and *E. durans*; however, the gene Na+/H+ antiporter (*nhaC*) that usually follow *tyrP* is missing. In addition, BLAST analysis revealed the presence of additional genes encoding for decarboxylase and permease in the genome of several *E. mundtii* strains. It is speculated the occurrence of a duplication event and the acquisition of different specificity for these enzymes that deserves further investigations.

## Introduction

Tyramine is a biogenic amine (BA) deriving from tyrosine decarboxylation and can have severe acute effects if ingested in excessive amounts with food, consisting in peripheral vasoconstriction, increased cardiac output, accelerated respiration, elevated blood glucose and release of norepinephrine, symptoms known also as ‘cheese reaction’ (Shalaby, 1994; McCabe‐Sellers *et al*., 2006; Marcobal *et al*., 2012). Tyrosine decarboxylase, the enzyme responsible for tyramine production, can use as substrate also phenylalanine, producing 2‐phenylethylamine, whose adverse effects are similar to tyramine (Marcobal *et al*., 2006).

In general, the amino acid decarboxylation leading to BA formation provides metabolic energy and/or resistance against acid stress (Molenaar *et al*., 1993; Fernández and Zúñiga, 2006; Pereira *et al*., 2009). The microorganisms responsible for tyramine accumulation in foods belong mainly to the group of lactic acid bacteria (LAB) (Marcobal *et al*., 2012). Among LAB, species belonging to the genus *Enterococcus* are recognized as the most frequent and intensive tyramine producers (Leuschner *et al*., 1999; Suzzi and Gardini, 2003; Ladero *et al*., 2012).

Due to their salt and pH tolerance, and to their ability to grow over a wide temperature range, enterococci are isolated from different habitats and are often contaminants in food of animal origin, such as cheese and sausages (Giraffa, 2003; Franz *et al*., 2011). In spite of their homolactic metabolism, their potential role in cheese ripening and their ability to produce bacteriocins (Beshkova and Frengova, 2012; Fontana *et al*., 2015), enterococci have a controversial status and they are often considered at the crossroad of food safety (Franz *et al*., 1999). In fact, this group is considered as indicator of the hygienic quality of raw material and food, as well as marker of faecal contamination (Leclerc *et al*., 1996). In addition, virulence factors can be present (Foulquié Moreno *et al*., 2006; Hollenbeck and Rice, 2012) and they can act as opportunistic human pathogens frequently associated with nosocomial infections due to their antibiotic resistance with a high capacity to disseminate this resistance to other microorganisms (Giraffa, 2002; Klein, 2003; Rossi *et al*., 2014). Furthermore, they are strong tyramine producers and this ability has been deeply exploited in *Enterococcus faecalis* (in which tyramine production is considered a species trait), *Enterococcus faecium* and *Enterococcus durans* (Linares *et al*., 2009; Ladero *et al*., 2012; Bargossi *et al*., 2015a,b). For this reasons, the presence of enterococci has been put in relation with the presence of tyramine in several fermented foods, such as fermented sausages (Gardini *et al*., 2008), cheeses (Linares *et al*., 2011) and wine (Pérez‐Martín *et al*., 2014). The enterococcal species most frequently isolated from fermented foods are *E. faecalis* and *E. faecium*, and also *E. durans, Enterococcus gallinarum, Enterococcus casseliflavus, Enterococcus hirae* can be found in food matrices (Franz *et al*., 2003; Giraffa, 2003; Foulquié Moreno *et al*., 2006; Corsetti *et al*., 2007; Komprda *et al*., 2008). Recently, also *E. mundtii* has been isolated from the food chain; it is a non‐motile, yellow‐pigmented enterococcus infrequently associated to human infection (Collins *et al*., 1986; Higashide *et al*., 2005). Strains of *E. mundtii* have been isolated from soy and cereals (Todorov *et al*., 2005; Corsetti *et al*., 2007), water (Moore *et al*., 2008; Graves and Weaver, 2010; Furtula *et al*., 2013), soil (Collins *et al*., 1986; Bigwood *et al*., 2012) and forage grass or silage, in which this species is often the predominant among enterococci (Muller *et al*., 2001; Ni *et al*., 2015). It has also been isolated from animals (Collins *et al*., 1986; Espeche *et al*., 2014) and from food (Vera Pingitore *et al*., 2012; Schöbitz *et al*., 2014). This species has been deeply studied in relation to the bacteriocin produced, among which mundticine (De Kwaadsteniet *et al*., 2005; Todorov *et al*., 2005; Corsetti *et al*., 2007; Feng *et al*., 2009; Vera Pingitore *et al*., 2012; Espeche *et al*., 2014).

Recently, the genome of *E. mundtii* QU 25, an efficient l‐lactic acid‐producing bacterium isolated from ovine faeces, has been completely sequenced (Shiwa *et al*., 2014), and comparative analysis of the genetic content of this species with respect to other representative enterococcal species of diverse origins was conducted (Repizo *et al*., 2014). Despite to those recent acquisitions, scarce information is available about *E. mundtii* tyraminogenic potential. Trivedi *et al*. (2009) carried out a study testing the ability to decarboxylate tyrosine in several enterococci isolated from different foodstuff. Regarding *E. mundtii*, four of five strains isolated from meat products and six of 12 isolated from vegetables and fruits possessed this ability. Also Kalhotka *et al*. (2012) found an *E. mundtii* strain able to produce tyramine and agmatine. This latter amine derives from the decarboxylation of arginine and can be transformed in putrescine by a specific deiminase (Linares *et al*., 2015).

In this research, the tyramine and 2‐phenylethylamine accumulation by two *E. mundtii* strains isolated from grass silage was studied during their growth in a rich medium. In addition, information on the genetic basis of the tyraminogenic potential of *E. mundtii* were obtained analysing the expression of the tyrosine decarboxylase (*tyrDC*) gene, the sequence of *tyrDC* and tyrosine permease (*tyrP*) genes, and the genetic organization of the TDC operon region.

## Results and discussion

### Tyramine‐positive enterococci

**Figure 1:**
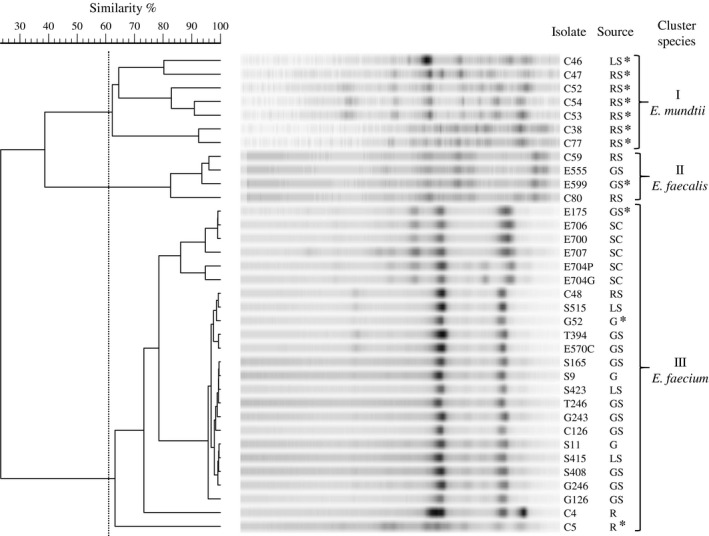
UPGMA dendrogram derived from RAPD‐PCR‐fingerprinting patterns of all the 35 isolates using the primer 1254. Code and source of the isolates are indicated on the right‐hand side of the figure. The vertical dotted line indicates the 60% similarity level that delineates the species *E. mundtii* (cluster I), *E. faecalis* (cluster II) and *E. faecium* (cluster III). Isolates marked with * were identified by phenylalanyl‐tRNA synthase α‐subunit (*pheS*) gene sequence analysis. G: maize grain; GS: maize grain silage; LS: lucerne silage; M: whole crop maize; MS: whole crop maize silage; R: ryegrass; RS: ryegrass silage; SC: starter cultures for silage.

In the first part of the research, 35 isolates of coccal LAB, originating from different agricultural foodstuffs (Fig. 1) and positive for the production of tyramine according to the method of Bover‐Cid and Holzapfel medium (Bover‐Cid and Holzapfel, 1999) were considered. These isolates were presumptively identified as enterococci based on their physiological and morphological characteristics (von Wright and Axelsson, 2012). They were cocci, Gram‐positive, catalase‐negative, non‐spore‐forming and occurring both as single cells and in chains. They were able to growth at 10°C and 45°C, at pH 4.4 and 9.6, and in the presence of 6.5% of NaCl.

To confirm the decarboxylase activity revealed by the Bover‐Cid and Holzapfel medium, the occurrence of the gene *tyrDC*, coding for tyrosine decarboxylase (TDC), was examined. A *tyrDC* gene fragment was amplified according to Torriani *et al*. (2008). For all the 35 isolates, the 336 bp amplicon was obtained, confirming their tyraminogenic potential.

Successively, RAPD‐PCR fingerprinting technique with the primer 1254 (Table 1) was applied to investigate the genetic diversity of the strains. This molecular typing method has proved to be reliable, discriminative and suitable for the study of a large number of strains in short time (Vancanneyt *et al*., 2002). The primer 1254 generated reproducible RAPD‐PCR fingerprints thanks to an accurate standardization of all the PCR and electrophoresis conditions. The reproducibility of PCR assays and running conditions, estimated by analysis of duplicate DNA extracts of several strains, was higher than 90%. Cluster analysis of the RAPD‐PCR fingerprints grouped the 35 isolates in three clusters (Fig. 1). Seven strains, all originated from ryegrass silage except one (C46), were grouped in the cluster I, four strains from ryegrass and maize grain silages belonged to the cluster II and, finally, 24 strains from different foodstuffs were clustered in the group III.

**Table 1:** Primers used in this study in RAPD‐PCR, RT‐qPCR and conventional PCR reactions and expected amplicon size

PCR type	Target	Primer code	Sequence (5′‐3′)	Amplicon (pb)	Reference
RAPD‐PCR	Arbitrary DNA sequences	1254	CCG CAG CCA A	Variable	Akopyanz *et al*. (1992)
RT‐qPCR	*tyrDC*	TYR3f	CGT ACA CAT TCA GTT GCA TGG CAT	171	Torriani *et al*. (2008)
		TYR4r	ATG TCC TAC TTC TTC TTC CAT TTG		
Conventional	*tyrDC*	DEC5	CGT TGT TGG TGT TGT TGG CAC NAC NGA RGA RG	350	
		DEC3	CCG CCA GCA GAA TAT GGA AYR TAN CCC AT		
	*pheS*	pheS‐21‐F	CAY CCN GCH CGY GAY ATG C	455	Naser *et al*. (2005)
		pheS‐22‐R	CCW ARV CCR AAR GCA AAR CC		
	*tyrS/tyrDC*	TyrS‐F1	GGA GCT ATA AGT ATT AAC GGT GA	940	Bargossi *et al*. (2015a)
		Tdc‐R1	GAT TT(A/G) ATG TT(A/G) CG(G/C) GCA TAC CA		
	*tyrDC*	Tdc‐F2	CAA ATG GAA GAA GAA GT(A/T) GGA	1340	
		Tdc‐R2	CC(A/G/T) GCA CG(G/T) T(C/T)C CAT TCT TC		
	*tyrDC*/*tyrP*	Tdc‐F3	CCA GA(C/T) TAT GGC AA(C/T) AGC CCA	788	
		TyrP‐R3	CCT AAA GTA GAA GC(A/G) ACC AT		
	*tyrP*	TyrP‐F4	TGG GTG CAA ATG TTC CCA GG	940	
		TyrP‐R4	ACC (A/G)AT TCG (A/G)TA AGG ACG		
	*tyrP*/*nhaC‐2*	TyrP‐F5	(A/T)CT GCT TGG GT(A/T) ACT GGA CC	na	
		NhaC‐R5	CAT (C/T)GC AT(C/T) (A/G)T(C/T) GAA TCC AAG		

na, no amplicon.

For each cluster, some representative isolates were chosen to proceed with their identification at the species level by the *pheS* gene analysis. Indeed, this gene is considered a reliable genomic marker for differentiating the species within the genus *Enterococcus*, and it was demonstrated to be much more discriminatory than 16S rRNA (Naser *et al*., 2005). The *pheS* gene has a high degree of homogeneity among strains of the same enterococcal species (at least 97% sequence similarity), whereas distinct species reveal at maximum 86% gene sequence similarity. The *pheS* partial gene sequence data obtained indicated that the strains C46, C53 and C77, grouped in the cluster I, can be assigned to the species *E. mundtii* (99–100% identity), the strain E599 (cluster II) to *E. faecalis* (100% identity), while the strains E175, G52 and C5 (cluster III) to *E. faecium* (100% identity). After that, the analysis of the *pheS* gene was extended to all the isolates of cluster I, thus confirming their belonging to the *E. mundtii* species.

These results confirmed the tyrosine decarboxylase potential of *E. faecalis* and *E. faecium*, the stronger tyramine producers (Aymerich *et al*., 2006; Bonetta *et al*., 2008; Gardini *et al*., 2008; Ladero *et al*., 2012; Marcobal *et al*., 2012). On the other hand, tyramine production is considered a species characteristic of *E. faecalis* (Ladero *et al*., 2012). In addition, the tyraminogenic potential of *E. durans* has been deeply studied (Fernández *et al*., 2007; Linares *et al*., 2009). Regarding *E. mundtii*, scarce are the studies on their capability to accumulate tyramine and the genetic aspects involved in its accumulations. Kalhotka *et al*. (2012) investigated the decarboxylase activity of enterococci isolated from goat milk and found that all of the tested strains, identified as *E. mundtii, E. faecium* and *E. durans,* showed significant tyrosine and arginine decarboxylase activity, in relation to temperature and duration of cultivation. In addition, Trivedi *et al*. (2009) studied the ability to decarboxylate tyrosine in many enterococcal strains isolated from different foodstuffs and found that more than 90% of isolates showed the presence of the gene *tyrDC*. In particular, these authors found that 10 of 17 *E. mundtii* strains were tyramine producers. These preliminary studies indicated the occurrence of tyramine‐producing *E. mundtii* strains, but did not highlight the tyraminogenic potential of this species. Moreover, the molecular aspects involved in the tyramine biosynthesis have not yet studied in depth. For this reason, two of the *E. mundtii* strains considered here were chosen as targets for investigating their tyramine accumulation capability and tyrosine metabolism. In particular, the two strains C53 and C46 were selected on the basis of their different origin and genetic diversity. Indeed, these strains have limited genetic similarity, belonging to different RAPD‐PCR subclusters, as shown in Fig. 1; in addition, C53 was the sole *E. mundtii* strain of the collection that originated from lucerne silage.

### Growth parameters and tyramine production of *Enterococcus mundtii* strains

**Figure 2:**
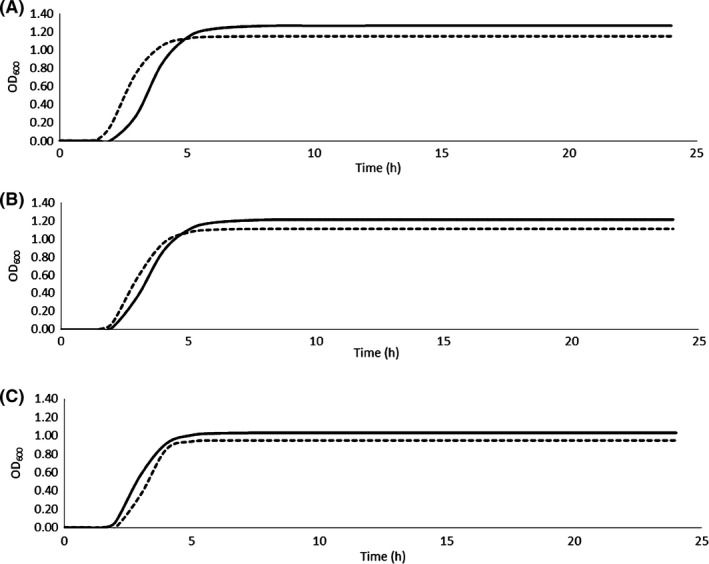
Growth curves of *E. mundtii* C46 (A) and *E. mundtii* C53 (B) obtained according to the Gompertz parameters reported in Table 2. The growth was obtained in BHI not added (solid line) or added (dotted line) with tyrosine. As a comparison, also the growth curves obtained under the same conditions for the strain *E. faecalis* EF37 (C) are reported, according to the data of Bargossi *et al*. (2015b).

The growth of the strains *E. mundtii* C46 and C53 was monitored by measuring the OD_600_ increase in BHI medium added or not with tyrosine. The OD_600_ changes were modelled with the Gompertz equation (Zwietering *et al*., 1990) and the estimates of the parameters are reported in Table 2. All the parameters were characterized by a high significance (*P* ≤ 0.05). Both the strains reached the maximum value of OD_600_ (*A*), ranging between 1.11 and 1.27, after 6–8 h incubation at 37°C. The curves presented a very short lag phase (*λ*), followed by a sharp increase of OD_600_. As far as *A* and *λ*, no marked differences were found among the two strains, while *E. mundtii* C53 presented a lower maximum OD_600_ increase rate in exponential phase (*μ*_max_). Moreover, the addition of tyrosine generally determined lower values of *A*, higher values of *μ*_max_ and a shorter lag phase. Table 2 reports also the cell counts detected at beginning of the stationary phase. The models obtained are graphically represented in Fig. 2, which reports the growth curves in the first 24 h of incubation. As a reference, the same figure shows also the growth curves obtained under the same conditions by Bargossi *et al*. (2015b) for *E. faecalis* EF37, a strong tyramine producer (Gardini *et al*., 2008), which exhibited analogous behaviours.

**Table 2:** Gompertz equation parameters for enterococcal growth measured as OD_600_. *R* ^2^ is given as diagnostics of the regression. The maximum cell concentrations (expressed as log CFU ml^−1^) at the beginning of the stationary phase is reported. The standard deviation is reported within parentheses

Strain	Cultural medium	Gompertz equation parameters^a^			*R* ^2^	Maximum cell concentration
		*A*	*μ*_max_	*λ*		
C46	BHI + tyr^b^	1.153 (± 0.029)	0.635 (± 0.079)	1.771 (± 0.119)	0.994	9.09 (± 0.04)
	BHI	1.269 (± 0.036)	0.615 (± 0.077)	2.556 (± 0.132)	0.994	9.06 (± 0.01)
C53	BHI + tyr	1.113 (± 0.037)	0.594 (± 0.101)	2.024 (± 0.177)	0.990	9.01 (± 0.02)
	BHI	1.215 (± 0.028)	0.563 (± 0.060)	2.345 (± 0.121)	0.996	8.97 (± 0.05)

a *A*: maximum OD_600_ value reached; *μ*_max_: maximum OD_600_ increase rate in exponential phase (OD_600_/h); *λ*: lag phase duration (h).

b BHI broth plus 1 g l^−1^ tyrosine.

The production of tyramine by *E. mundtii* C46 and C53 during their growth in BHI, added or not with the precursor, is shown in Table 3, which reports also the accumulation of 2‐phenylethylamine. Also in this case, the data already available for *E. faecalis* EF37 (Bargossi *et al*., 2015b) are reported. It is well known that enterococci can decarboxylate phenylalanine producing 2‐phenylethylamine through the activity of the same decarboxylase. The characteristics of this BA are very similar to tyramine, but it is produced with a lower efficiency (Marcobal *et al*., 2006).

**Table 3:** OD_600_ and tyramine (TYR) and 2‐phenylethylamine (2‐PHE) production by *E. mundtii* C53 and C46 during their growth in BHI, added or not with 1% tyrosine. It is also reported the production of TYR and 2‐PHE by *E. faecalis* EF37 strain (adapted from Bargossi *et al*., 2015b). The standard deviations are reported within parentheses

Time (h)	*E. mundtii* C53						*E. mundtii* C46						*E. faecalis* EF37^a^					
	BHI			BHI + 0.1% tyrosine			BHI			BHI + 0.1% tyrosine			BHI			BHI + 0.1% tyrosine		
	OD_600_ b	TYR (mg l^−1^)	2‐PHE (mg l^−1^)	OD_600_	TYR (mg l^−1^)	2‐PHE (mg l^−1^)	OD_600_	TYR (mg l^−1^)	2‐PHE (mg l^−1^)	OD_600_	TYR (mg l^−1^)	2‐PHE (mg l^−1^)	OD_600_	TYR (mg l^−1^)	2‐PHE (mg l^−1^)	OD_600_	TYR (mg l^−1^)	2‐PHE (mg l^−1^)
2	0.000	8.35 (± 0.41)	–c	0.000	20.31 (± 0.32)	–	0.004	7.14 (± 0.19)	–	0.167	15.66 (± 0.65)	–	0.059	n.d.^d^	n.d.	0.000	n.d.	n.d.
3	0.367	21.30 (± 1.12)	–	0.575	42.18 (± 1.05)	–	0.279	21.56 (± 0.72)	–	0.748	72.89 (± 2.04)	–	0.575	n.d.	n.d.	0.359	n.d.	n.d.
4	0.865	32.16 (± 1.84)	–	0.953	64.88 (± 1.54)	–	0.846	36.59 (± 0.08)	–	1.047	130.34 (± 2.56)	–	0.913	n.d.	n.d.	0.851	n.d.	n.d.
5	1.103	46.29 (± 1.70)	–	1.073	93.59 (± 2.32)	–	1.139	61.37 (± 1.81)	–	1.128	189.87 (± 3.63)	–	1.004	n.d.	n.d.	0.936	n.d.	n.d.
8	1.212	72.25 (± 2.31)	–	1.112	221.25 (± 5.48)	–	1.267	97.55 (± 2.50)	4.80 (± 0.06)	1.153	396.36 (± 3.68)	–	1.029	11.65 (± 1.75)	39.67 (± 1.71)	0.947	503.75 (± 6.16)	85.21 (± 2.12)
24	1.215	101.71 (± 3.44)	11.77 (± 0.48)	1.113	508.88 (± 5.93)	4.07 (± 0.80)	1.269	112.33 (± 4.32)	33.24 (± 1.24)	1.153	630.09 (± 4.75)	6.72 (± 0.74)	1.029	90.97 (± 6.71)	177.10 (± 5.46)	0.947	536.16 (± 4.32)	295.61 (± 5.75)
48	1.215	116.73 (± 6.78)	32.52 (± 0.87)	1.113	691.44 (± 8.49)	6.91 (± 0.22)	1.269	121.42 (± 0.96)	63.21 (± 3.09)	1.153	770.35 (± 7.06)	14.84 (± 0.95)	1.029	69.64 (± 2.93)	213.79 (± 7.25)	0.947	551.40 (± 4.43)	405.80 (± 6.17)
72	1.215	129.12 (± 4.09)	56.26 (± 0.94)	1.113	757.43 (± 3.69)	24.59 (± 0.65)	1.269	127.57 (± 1.24)	91.00 (± 2.16)	1.153	781.50 (± 5.83)	43.46 (± 1.92)	1.029	68.30 (± 4.88)	262.45 (± 6.87)	0.947	513.94 (± 5.65)	428.50 (± 4.91)
96	1.215	134.15 (± 2.11)	75.63 (± 1.68)	1.113	766.57 (± 9.91)	20.55 (± 0.71)	1.269	129.46 (± 1.68)	108.56 (± 3.82)	1.153	797.28 (± 9.95)	44.94 (± 2.16)	1.029	n.d.	n.d.	0.947	n.d.	n.d.

a Adapted from Bargossi *et al*. (2015b).

b Optical density at the different sampling time as predicted by the Gompertz model (Table 2).

c Under the detection limit (0.5 mg l^−1^).

d n.d., not determined.

In all the tested conditions, the two *E. mundtii* strains were able to accumulate tyramine independently on the addition of tyrosine. In fact, the decarboxylase activity was detected also in the medium not supplemented with tyrosine, because BHI contains amino acid sources (proteins and peptides) among which precursors for TDC. This observation was previously reported by Bargossi *et al*. (2015b) for *E. faecalis* and *E. faecium* grown in the media BHI and Bover‐Cid and Holzapfel.

The data showed that the two *E. mundtii* strains began to produce tyramine after 2 h from the inoculum, both in the presence and in the absence of the precursor, and they continued to gradually accumulate tyramine during their stationary phase. In all the conditions, the maximum tyramine concentration was reached after 48 h for the strain C46 and after 72 h for the strain C53. However, the final amount of tyramine was similar for both the strains. In fact, it did not exceed 135 mg l^−1^ in BHI medium, while in the presence of tyrosine, the final amount of tyramine was about 767 and 797 mg l^−1^ for the strains C53 and C46 respectively. As reported in Table 3, Bargossi *et al*. (2015b) found that *E. faecalis* EF37 under the same conditions after 8 h reached the maximum tyramine concentration in the presence of tyramine added. The *E. mundtii* strains showed a slower tyramine production kinetics, but the final amount was significantly higher than *E. faecalis* EF37 (approximately 500 mg l^−1^). In the absence of tyrosine added, the strain *E. mundtii* C46 was characterized by a faster tyramine accumulation in BHI. The major differences between *E. faecalis* EF37 and the *E. mundtii* strains were in the ability to accumulate 2‐phenylethylamine, which was dramatically higher in *E. faecalis*. These amounts were higher than those reported by Liu *et al*. (2013) who, testing the tyraminogenic potential of *E. faecalis* strains from water‐boiled salted duck, found concentrations of tyramine lower than 330 mg l^−1^ in MRS broth added with 0.1% tyrosine.

The two *E. mundtii* strains were also able to decarboxylate phenylalanine leading to the production of 2‐phenylethylamine (Table 3). This BA was accumulated only after 24 h of growth for the strain C53, while C46 began to produce this compound already after 8 h in the absence of tyrosine. The 2‐phenylethylamine accumulation increased during subsequent incubation and reached its maximum level after 72 h with amended tyrosine and after 96 h without this amino acid. Moreover, the production of 2‐phenylethylamine was higher when tyrosine was not added to the growth medium. Indeed, in this case, concentrations of about 76 and 109 mg l^−1^ for *E. mundtii* C53 and C46, respectively, were reached, compared with concentrations lower than 45 mg l^−1^ in BHI when tyrosine was added to the medium. Interestingly, however, the accumulation of this BA became relevant when the tyramine concentration reached its maximum level (independently on the addition of the precursor). In any case, the amount of this BA was lower than that accumulated by *E. faecium* FC12 and *E. faecalis* EF37 (more than 400 mg l^−1^) grown in the same medium (Bargossi *et al*., 2015b). These findings could reflect the lower efficiency of the *E. mundtii* TDC for phenylalanine decarboxylation and could indicate that these amounts of tyramine can lower or inhibit further decarboxylase activities in the tested strains.

The continue tyramine accumulation until late stationary growth phase observed in this research could represent an advantage for the microorganism against acidification during the fermentation process and growth. In fact, the decarboxylation of amino acids has been indicated as a mechanism through which LAB and human pathogenic bacteria can resist acidic conditions (Lund *et al*., 2014; Romano *et al*., 2014) and this protective effect seems to be mediated via the maintenance of intracellular pH (Perez *et al*., 2015). The same role in the maintenance of pH homoeostasis in acidic environment has been also described in *E. durans* (Linares *et al*., 2009) and *E. faecium* (Marcobal *et al*., 2006).

### Time‐course of *tyrDC* gene expression

Table 4 reports the *tyrDC* gene expression data obtained for *E. mundtii* C46 and C53 by RT‐qPCR during 72 h growth in BHI supplemented or not with tyrosine. The *tyrDC* gene expression data previously obtained for *E. faecalis* EF37 by Bargossi *et al*. (2015b) are also reported as a reference.

**Table 4:** Tyrosine decarboxylase (*tyrDC)* gene expression data for *E. mundtii* C46 and C53 grown in BHI added or not with 0.1% tyrosine during 72 h, as determined by RT‐qPCR. The *tyrDC* gene expression data for *E. faecalis* EF37 is also reported (adapted from Bargossi *et al*., 2015b). The standard deviation is reported within parentheses

Strain	Cultural medium	Log (copies/μg cDNA) at time (h)							
		2	3	4	5	8	24	48	72
C46	BHI	3.4 (± 0.06)	3.0 (± 0.03)	2.5 (± 0.03)	2.7 (± 0.30)	3.1 (± 0.004)	2.9 (± 0.14)	3.0 (± 0.13)	2.3 (± 0.83)
	BHI + tyr^a^	2.9 (± 0.002)	3.5 (± 0.06)	4.6 (± 0.05)	4.1 (± 0.13)	2.5 (± 0.03)	3.1 (± 0.11)	1.6 (± 0.04)	1.6 (± 0.13)
C53	BHI	2.7 (± 0.31)	3.0 (± 0.39)	3.3 (± 0.07)	2.3 (± 0.22)	2.6 (± 0.07)	2.1 (± 0.03)	2.0 (± 0.03)	1.3 (± 0.27)
	BHI + tyr^a^	2.2 (± 0.22)	3.0 (± 0.09)	4.2 (± 0.21)	3.7 (± 0.19)	2.2 (± 0.03)	2.0 (± 0.04)	1.4 (± 0.14)	1.1 (± 0.14)
EF37	BHI	5.08 (± 0.02)	n.d.^b^	4.87 (± 0.01)	n.d.	5.22 (± 0.05)	2.42 (± 0.07)	2.81 (± 0.03)	1.01 (± 0.29)
	BHI + tyr^a^	4.79 (± 0.06)	n.d.	6.11 (± 0.02)	n.d.	5.03 (± 0.05)	4.15 (± 0.05)	3.38 (± 0.03)	4.10 (± 0.12)

a BHI broth plus 1 g l^−1^ tyrosine.

b n.d., not determined.

In general, the *tyrDC* gene expression time‐course did not differ considerably between the two *E. mundtii* strains, even if the values found for the strain C53 were averagely lower. These data are in compliance with the phenotypic behaviour of the two analysed *E. mundtii* strains, as they showed similar trends in the accumulation of tyramine and phenylethylamine, and produced comparable final levels of these BAs in the different tested conditions.

In the medium without tyrosine, a high value of transcript (about 3 log copies/μg cDNA) was already observed after 2 h (early exponential phase), probably due to the strong residual effect of the precursor present in the pre‐cultivation medium. The amount of *tyrDC* transcript remained rather stable throughout all the period monitored. The addition of the precursor affected considerably the *tyrDC* expression level depending on the growth phase. Indeed, the expression of *tyrDC* increased rapidly, peaked (> 4 log copies/μg cDNA) at 4 h during the exponential phase of growth, when the highest number of cells for ml was reached. After 8 h, the gene expression decreased progressively until the end of the 72‐h period monitored.

As notice above, the *E. mundtii* strains were able to accumulate greater amounts of BAs than that of other previously studied enterococcal strains *E. faecalis* EF37 and *E. faecium* FC12 under the same conditions (Bargossi *et al*., 2015b). However, the maximum *tyrDC* gene copies number of *E. mundtii* C46 and C53, obtained after 4 h growth in BHI with tyrosine, did not reach the value found for *E. faecalis* EF37 (6.1 log copies/μg cDNA) in the same conditions. The expression trend of the *E. mundtii* strains in BHI without tyrosine was more similar to that of *E. faecium* FC12 which presented a rather constant *tyrDC* transcript level during the entire incubation period. However, in BHI added with tyrosine, the expression profile differed between the *E. mundtii* strains and *E. faecium* FC12 because the *tyrDC* gene transcript reached the maximum level in the exponential (4 h) and in the stationary phase (24 h), respectively, when the highest cell number of 9 log CFU ml^−1^ was detected for all these strains.

### Analysis of the TDC operon region

**Figure 3:**
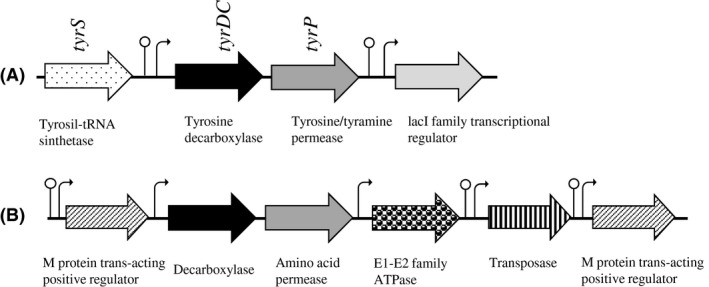
(A) Organization of the TDC operon in the strain *E. mundtii* QU 25 (GCA_000504125.1); (B) genome fragment encoding for an additional PLP‐dependent decarboxylase, an APC family amino acid transporter and a cation transporter E1‐E2 family ATPase; upstream is recognized as a M protein trans‐acting positive regulator and downstream as an ISEfa11 (ISL3 family) transposase, followed by an additional M trans‐acting positive regulator gene.

The characteristics of the TDC operon region involved in tyramine production have been described in several tyraminogenic bacterial strains, including enterococci (Connil *et al*., 2002; Lucas *et al*., 2003; Coton *et al*., 2004; Fernández *et al*., 2004; Marcobal *et al*., 2012; Bargossi *et al*., 2015a). However, the molecular knowledge of this region for *E. mundtii* is extremely scarce. Therefore, an investigation was carried out to determine the DNA and amino acid sequences of the *E. mundtii* C46 tyramine production‐associated genes and the genetic organization of the TDC operon region, considering also the available genome sequencing data. In particular, the region downstream the gene *tyrS* including the genes *tyrDC* and *tyrP*, which encode for the tyrosine decarboxylase and the tyrosine/tyramine permease, respectively, was amplified and sequenced. Indeed, the gene Na^+^/H^+^ antiporter (*nhaC*), that usually follow *tyrP* in the TDC operon of several tyramine‐producing LAB, such as *E. faecalis*,* E. faecium* and *L. brevis* (Marcobal *et al*., 2012; Bargossi *et al*., 2015a) was not recognized by PCR performed with the primers covering the intergenic region between *tyrP* and *nhaC*. Such gene organization was found also in the fully sequenced and assembled genome of *E. mundtii* QU 25 (Shiwa *et al*., 2014) (GCA_000504125.1) that shows a lacI family transcriptional regulator gene downstream *tyrP* (Fig. 3A).

BLASTN analysis of the 3677 bp nucleotide sequence of the *E. mundtii* C46 TDC operon region showed the best overall identity of 99% (3673/3677 nt) with that of *E. mundtii* QU 25. High levels of DNA sequence identity (> 80%) were also found for several strains belonging to other enterococcal species: *E. hirae* ATCC 9790 (1884/2282, 83%), *E. durans* KLDS 6.0930 and KLDS 6.0933 (1876/2285, 82%), and *E. faecium* Aus0085, NRRL B‐2354, Aus0004, DO, and T110 (1877/2286, 82%). On the contrary, lower sequence identity (76%) was achieved for strains belonging to the species *E. faecalis* (e.g. ATCC 29212 and V583). Putative promoter and terminator were found upstream the start codon of the genes *tyrDC* (Fig. 3A), but not in the short intergenic sequence before the gene *tyrP*, suggesting that these two genes are probably co‐transcribed, as already showed for other species, such as *E. faecalis* and *L. brevis* (Marcobal *et al*., 2012).

Surprisingly, BLASTN analysis discovered in the genome of *E. mundtii* QU 25 (Shiwa *et al*., 2014), the presence of another region constituted by two genes similar to *tyrDC* and *tyrP*. These genes showed lower sequence identity values, 69% and 64%, respectively, with those present in the TDC operon. The genetic organization of the genomic segment that includes these two genes is shown in Fig. 3B. This additional portion was also recovered in the genome of other enterococcal strains, such as *E. hirae* ATCC 9790, *E. faecium* NRRL B‐2354, *E. durans* KLDS6.0930 and KLDS6.0930. However, in these strains a further putative amino acid permease was annotated between the tyrosine permease and the cation transporter E1‐E2 family ATPase. The presence of a gene associated to a transposase after the ATPase encoding gene in *E. mundtii* QU 25 (Shiwa *et al*., 2014) is of particular interest, as it could be involved in spontaneous events of gene duplication or horizontal transfer.

BLASTX analysis and comparison of the deduced amino acid sequences of *E. mundtii* C46 TDC operon region were also carried out. The translated nucleotide sequence generated two proteins in the frame +1 and +2 respectively. The first one showed the highest identity with a tyrosine decarboxylase (BAO05941.1) of *E. mundtii* QU 25 (624/624 nt, 100%) and *E. mundtii* CRL35 (616/624 nt, 99%) and decreasing identity (90% to 71%) with decarboxylases from other species of the genus *Enterococcus*. On the contrary, lower similarity (61% to 9%) was found with the additional PLP‐dependent decarboxylase detected with BLASTN analysis. The second protein presented a putative conserved domain associated to a putative glutamate/gamma‐aminobutyrate antiporter (TIGR03813). This sequence showed 100% identity with the amino acid permease family protein of *E. mundtii* QU 25 and *E. mundtii* ATCC 883, and decreasing identity with the amino acid permeases of other species of the genus *Enterococcus*. Also in this case, lower identity (58–60%) was found with the additional amino acid permease detected with BLASTN analysis.

These sequence analysis results taken together indicated the presence in the *E. mundtii* genome of a TDC operon with a classical genetic organization (i.e. *tyrS*,* tyrDC* and *tyrP*) and provided evidences for a new additional copy consisting of three ORF. According to Lynch and Conery (2000), duplications of a genome segments have been thought to be a primary source of material for the origin of evolutionary novelties, including new gene functions and expression patterns. Therefore, the additional copy may acquire a novel, beneficial function and become preserved by natural selection, with the other copy retaining the original function. Recently, Bargossi *et al*. (2015a) described the compromised tyrosine decarboxylase activity of the strain *E. faecium* FC643 due to a codon stop in the translated *tyrDC* sequence. However, this strain showed a slow and reduced production of tyramine, and not 2‐phenylethylamine, probably due to the presence of the additional enzyme with different substrate specificity and regulation mechanism respect to the decarboxylase encoded by the gene *tyrDC* of the TDC operon.

As regards *E. mundtii*, it can be supposed that all the genes in the two operon regions detected are expressed and produce functional products. As BLAST analysis revealed that the primer pairs DEC5/DEC3 and TYR3f/TYR4r used in this study were able to match conserved regions on both the putative *tyrDC* genes present in the *E. mundtii* QU25 genome, new target‐specific primers have to be designed to detect and analyse the contribute of the additional genes to the overall tyraminogenic potential of *E. mundtii*. Therefore, the role of the additional genes and proteins in the context of BA production needs further deep investigation.

## Conclusions

In this study, the capability of *E. mundtii* strains to accumulate tyrosine and 2‐phenylalanine in cultural media was assessed, and more information on the genetic basis of their tyraminogenic potential were obtained for the first time. The two strains considered here produced greater amounts of tyramine than those accumulated by other strains belonging to *E. faecium* and *E. faecalis* previously studied in the same conditions (Bargossi *et al*., 2015b). By contrast, their ability to decarboxylate phenylalanine was less enhanced if compared with the same strains. Likewise the other enterococcal strains, the expression analysis of the gene *tyrDC* showed that an excess of the precursor tyrosine affected the amount of the transcript during the exponential phase of growth, and that the amino acids fraction present in the medium also modulated the level of the transcript. The genetic organization as well as sequence identity levels of the genes *tyrDC* and *tyrP* indicated that the tyramine‐forming pathway in *E. mundtii* is similar to those in phylogenetically closer enterococcal species, such as *E. faecium*,* E. hirae* and *E. durans*; however, the gene Na^+^/H^+^ antiporter (*nhaC*) that usually follow *tyrP* is missing. Analysis of the available data on genome content and organization of *E. mundtii* QU 25 (Shiwa *et al*., 2014) and other *Enterococcus* strains revealed an unexpectedly presence of another region that includes two genes encoding for an additional PLP‐dependent decarboxylase and an amino acid permease. It is tempting to speculate that a duplication event occurred and the evolution of this redundant copy induced the acquisition of different specificity leading to the maintenance of both the functional copies. Thus, this discovery uncovers another level of complexity in the enterococcal BAs regulatory network. Further studies have to be performed to better explain the genetic and functional characteristics of these further enzymes and their correlation with tyrosine decarboxylating potential of enterococci. Moreover, regulation of decarboxylases and permeases at protein level has to be evaluated to verify if post‐translational mechanisms could affect and modulate enzymatic activities.

## Experimental procedures

### Characterization of the strains and screening procedure for tyramine production

In the present study, we used 35 cocci LAB isolates (Fig. 1), deposited in the bacterial culture collection of the Biotechnology Department of the Verona University. They were previously isolated from different fresh and ensiled forage crops (namely lucerne, ryegrass, maize), maize grain silage and starter cultures for silages, as shown in Fig. 1.

All isolates were maintained as culture stocks in 20% (w/v) glycerol at −80°C and grown aerobically in BHI Broth (Oxoid, Basingstoke, UK) at 37°C for 24 h, unless indicated otherwise.

The isolates were tested for morphological characteristics, Gram test, catalase test, growth in the presence of 6.5% NaCl, growth at 15 and 45°C and at pH 4.4 and 9.6, as well as for their homo or heterolactic fermentation.

The tyrosine decarboxylase activity of the isolates was evaluated using the screening plate method described by Bover‐Cid and Holzapfel (1999).

### TyrDC gene detection

Genomic DNA of tyramine‐positive isolates was obtained from 1 ml of overnight culture by using the Wizard Genomic DNA purification system (Promega Corporation, Madison, WI, USA), following the manufacturer's instructions. Isolates were assayed for the presence of the gene *tyrDC* by PCR analysis with the primers DEC5 and DEC3 (Table 1), following the conditions described previously (Torriani *et al*., 2008). PCR product was visualized on a 2% agarose gel.

### Randomly amplified polymorphic DNA (RAPD) analysis and identification of tyramine‐positive cocci

In order to genetically typify the 35 tyramine‐positive coccal strains, a preliminary RAPD‐PCR analysis was performed with the primer 1254 (Table 1). Conversion, normalization and numerical analysis of the patterns were performed by gelcompar 4.0 software (Applied Maths, Kortrijk, Belgium). A dendrogram was produced and major clusters with a cut‐off point of about 60% in the UMPGA (Unweighted Pair Group Method with Arithmetic Averages) clustering analysis similarity level was taken as representing a single cluster. Species identification was carried out by phenylalanyl‐tRNA synthase α‐subunit (*pheS*) gene sequence analysis (Naser *et al*., 2005). The *pheS* partial gene amplification was obtained with the primers pheS‐21‐F and pheS‐22‐R (Table 1). PCR conditions were set according to Naser *et al*. (2005) with exception that annealing temperature was 50°C. The expected amplicon (455 bp) was purified with the Wizard SV gel and PCR clean‐up system (Promega Corporation) and cloned with the cloning kit pGEMT‐easy vector system (Promega Corporation). Recombinant plasmids were sequenced at the GATC Biotech Ltd (Koln, Germany). Data were analysed with the Basic Local Alignment Search Tool (BLAST) provided by National Center for Biotechnology Information (NCBI) (http://blast.ncbi.nlm.nih.gov/Blast.cgi).

### Growth parameters of two *Enterococcus mundtii* strains and tyramine production

Two strains (C46 and C53), isolated from grass silage and identified as *Enterococcus mundtii*, were used for deeper investigations. The two considered enterococci were pre‐cultivated for 24 h at 37°C in BHI broth added with 1000 mg l^−1^ of tyrosine (Sigma‐Aldrich, Gallarate, Italy). After 24 h of pre‐cultivation, the microorganisms were inoculated, at a concentration of approximately 7 log CFU ml^−1^, in BHI broth, added or not with 1 g l^−1^ of tyrosine and incubated at 37°C for 72 h. The evaluation of the strain growth in BHI was performed by measuring the OD_600_ with a UV‐VIS spectrophotometer (Cary 60 UV‐Vis; Agilent Technologies, Santa Clara, CA, USA) with plastic cuvettes (1.5 ml) at defined times (1, 2, 3, 4, 5, 6, 7, 8, 24, 48, 72 and 96 h). The OD_600_ data were fitted with the Gompertz equation as modified by Zwietering *et al*. (1990).

$$y=k+Ae^{-e}\left[\left(\frac{\mu {}_{\max^{E}}}{A}\right)(\lambda -t)+1\right]$$

where *y* is the OD_600_ at time *t*,* A* represent the maximum OD_600_ value reached, μ_max_ is the maximum OD_600_ increase rate in exponential phase and *λ* is the lag time.

The maximum cell concentration reached was determined at the beginning of the stationary phase by plate counting enterococci onto BHI agar.

The BAs were determined after 2, 3, 4, 5, 8, 24, 48, 72 and 96 h of incubation. The cultures were centrifuged at 10 000 rpm for 10 min at 10°C, and the supernatants were used for BAs determination by HPLC after derivatization with dansyl‐chloride (Sigma‐Aldrich, Gallarate, Italy) according to Bargossi *et al*. (2015b). The quantification was performed according to Tabanelli *et al*. (2012) and the amount of tyramine and 2‐phenylethylamine was expressed as mg ml^−1^ by reference to a calibration curve obtained with standard solutions. The trials were always analysed in triplicate.

### RNA isolation, cDNA synthesis and RT‐qPCR assay

Two millilitre aliquots of *E. mundtii* cultures were centrifuged at 3000 rpm for 10 min and total RNA was isolated from the collected cell pellets according to Bargossi *et al*. (2015b). Total cDNA was synthesized from 1 μg of RNA using the ImProm‐IITM Reverse Transcriptase kit (Promega Corporation), following the manufacturer's recommendations.

The expression level of the gene *tyrDC* was analysed by a reverse transcription‐quantitative real time PCR (RT‐qPCR) assay with the primers TYR3f and TYR4r (Table 1); thermo cycler, reaction mixture and amplification programme were previously described in Torriani *et al*. (2008), as well as the procedure of the absolute quantification of the *tyrDC* copies number. Two independent biological replicates were performed for each trial and data were obtained from two technical replicates per sample.

### Analysis of the TDC operon region

The TDC operon fragments were obtained for *E. mundtii* C46 and C53 by PCR amplification with the partially degenerate primers reported in Table 1. PCR mixture was composed of 1× PCR buffer, 1.5 mM MgCl_2_, 200 nM dNTPs, 0.5 μM each primer and 50 ng DNA. Amplification programme comprised: 95°C for 5 min, 35 cycles at 94°C, 30 s; 56°C, 45 s; 72°C, 1 min and final extension at 72°C, 10 min. Amplicons were purified, cloned and sequenced as reported above. The partial TDC operon sequences of the strains *E. mundtii* C46 and C53 were submitted to the GenBank nucleotide database under the accession numbers KU870523 and KU870522 respectively.

Promoters prediction was carried out by BPROM, a bacterial sigma70 promoter recognition program (http://linux1.softberry.com/berry.phtml?topic=bprom%26group=programs%26subgroup=gfindb; Solovyev and Salamov, 2011). Putative Rho‐independent transcription terminators were predicted by the Arnold Finding Terminators (http://rna.igmors.u-psud.fr/toolbox/arnold/index.php).

Similar searches were performed with the BLAST programs available at the NCBI. Sequence alignments were carried out with the Clustal Omega analysis Tool Web Services from the EMBL‐EBI (Sievers *et al*., 2011).

### Statistical analysis

The growth model was fitted using the statistical package Statistica for Windows 6.1 (Statsoft Italia, Vigonza, Italy).

## Conflict of interest

None declared.
